# Gut Microbial Diversity in Female Patients With Invasive Mole and Choriocarcinoma and Its Differences Versus Healthy Controls

**DOI:** 10.3389/fcimb.2021.704100

**Published:** 2021-08-26

**Authors:** Xiaomei Liu, Xue Pan, Hao Liu, Xiaoxin Ma

**Affiliations:** Department of Obstetrics and Gynecology, Shengjing Hospital of China Medical University, Shenyang, China

**Keywords:** gestational trophoblastic neoplasia, invasive mole, choriocarcinoma, gut microbiota, microbiome

## Abstract

**Objective:**

To investigate variation in gut microbiome in female patients with invasive mole (IM) and choriocarcinoma (CC) and compare it with healthy controls.

**Methods:**

Fecal microbiome of 12 female patients with IM, 9 female patients with CC, and 24 healthy females were analyzed based on 16s rDNA sequencing. Alpha (α) diversity was evaluated using Shannon diversity index and Pielou evenness index, while beta (β) diversity was assessed using principle coordinate analysis (PCoA) of unweighted Unifrac distances. The potential functional changes of microbiomes were predicted using Tax4Fun. The relative abundance of microbial taxa was compared using Welch’s t test. The role of varied gut microbiota was analyzed *via* receiver operating characteristic (ROC) curve.

**Results:**

The α diversity and β diversity were significantly different between IM patients and controls, but not between CC patients and controls. In addition, the abundance of cancer-related genes was significantly increased in IM and CC patients. Notably, a total of 19 families and 39 genera were found to have significant differences in bacterial abundance. ROC analysis indicated that *Prevotella_7* may be a potential biomarker among IM, CC, and controls.

**Conclusion:**

Our study demonstrated that the diversity and composition of gut microbiota among IM patients, CC patients, and healthy females were significantly different, which provides rationale for using gut microbiota as diagnostic markers and treatment targets, as well as for further study of gut microbiota in gestational trophoblastic neoplasia (GTN).

## Introduction

Gestational trophoblastic diseases (GTDs), originated from trophoblast cells, are pregnancy-related diseases, including benign, partial, and complete hydatidiform moles (HM), and malignant moles [gestational trophoblastic neoplasia (GTN)] (Victoria Diniz and Sun, 2018; Silva et al., 2021). GTN is a member of GTDs and can be developed from partial or complete HM (Golfier et al., 2007; Lurain, 2010; Sun et al., 2016). Studies have shown that GTN can be formed by abnormal proliferation of trophoblast tissue after HM pregnancy or non-HM pregnancy (Seckl et al., 2013). Unfortunately, GTN can occur after any pregnancy event, including ectopic pregnancy and full-term pregnancy (Lurain, 2010). GTN is divided into invasive mole (IM), choriocarcinoma (CC), placental site trophoblastic tumor (PSTT), and epithelioid trophoblastic tumor (ETT) in accordance with the International Federation of Obstetrics and Gynecology (FIGO) scoring system (FIGO Committee on Gynecologic Oncology, 2009; Lurain, 2010). In most cases, GTN is usually diagnosed by monitoring human chorionic gonadotrophin (hCG) combined with histopathology (Lok et al., 2020). Progressively, specific tissue biomarkers have been increasingly used for differential diagnosis (Serino et al., 2012; Le Chatelier et al., 2013; Qian et al., 2020). An accurate diagnosis will contribute to the highest cure rate of up to 98% for CC and IM patients (Abu-Rustum et al., 2019). Therefore, novel markers and targets are essential for the diagnosis and treatment of GTN.

Gut microbiome plays an important role in human microbiome and health. Accumulating data have shown that alterations in gut microbiome contribute to the development, prognosis, and treatment of many diseases, including cancers (AlHilli and Bae-Jump, 2020). The abundance and diversity of gut microbiome have significant effects on carcinogenesis and immune response (McKenzie et al., 2021). Review reported by Chase et al. highlighted an important gap in the association between gut microbiome and gynecologic cancers (Chase et al., 2015). Gut microbiome altered host immunity by modulating multiple immune pathways, thus impacting cancer risk and treatment outcomes of various malignancies (Brestoff and Artis, 2013; Gopalakrishnan et al., 2018; McQuade et al., 2019). Previous studies have shown that a diverse gut microbiota with distinct composition improved anti-tumoral immune response by activating anti-tumoral T cells (Sivan et al., 2015; Herrera et al., 2019), suggesting that there may be a relationship between gut microbiome and GTN.

Unfortunately, the relationship between gut microbiome and GTN has not been reported. Therefore, in this study, the diversity and composition of gut microbiome was compared by 16s rDNA sequencing in fecal samples collected from 12 patients with IM, 9 patients with CC, and 24 healthy women, laying a foundation for further study of gut microbiome in GTN.

## Materials and Methods

### Sample Collection

The study was approved by the Medical Ethical Committee of Shengjing Hospital Affiliated to China Medical University on August 30, 2020. Upon completion of the questionnaire, all participants signed a written informed consent and retained a copy of the consent form. Subsequently, the patients were enrolled in the study. The Medical Ethics Committee approved the consent procedure. Fecal samples from 12 patients with IM (N = 12), 9 patients with CC (N = 9), and 24 healthy female controls (N = 24) were collected from our hospital between September 22, 2019, and August 30, 2020. Eligible women must be Chinese-speaking, have at least a primary school education, and have voluntarily agreed to participate in the study. Those who were taking psychiatric medications or had a history of chronic conditions were excluded from the study. The mean time from diagnosis to questionnaire completion was 5.13 months. Patients ranged in age from 21 to 69 years (mean age = 38.8 years). Samples were collected for study prior to any treatment. Fecal samples were collected by the patient after receiving the doctor’s notice, and sent to the laboratory within 15 min. The samples were stored at −80°C for use.

### DNA Extraction, PCR Amplification, and 16S rRNA Gene Sequencing

Microbial DNA was extracted using the HiPure Stool DNA Kit (Magen, Guangzhou, China) according to the protocol recommended by manufacturer. V3-V4 region of 16s rRNA genes was amplified by PCR with the primers 341-F, 5′-CCTACGGGNGGCWGCAG-3′ and 806-R, 5′-GGACTACHVGGGTATCTAAT-3′ (Guo et al., 2017), and the amplification procedure was as follows: Initial denaturation at 94°C for 2 min, followed by denaturation at 98°C for 10 s, annealing at 65°C for 30 s, and extension at 68°C for 30 s. This round was repeated for 30 cycles, followed by final extension at 68°C for 5 min. PCR reactions were performed in triplicate, and the reaction system was composed of 5 μl of 10 × KOD Buffer, 5 μl of 2 mM dNTPs, 3 μl of 25 mM MgSO_4_, 1.5 μl of each primer (10 μM), 1 μl of KOD Polymerase, and 100 ng of template DNA, with 50 μl in total. After amplification, the products were purified using the AxyPrep DNA Gel Extraction Kit (Axygen Biosciences, Union City, CA, USA) and quantified using ABI StepOnePlus Real-Time PCR System (Life Technologies, Foster City, USA). Purified products were pooled in equimolar and paired-end sequenced (PE250) on an Illumina platform according to the standard protocols. Raw reads have been deposited into the NCBI Sequence Read Archive (SRA) database, and all data sets have been accessible (accession number SRP313846).

### Sequence Data Processing

To get high-quality clean reads, raw reads containing more than 10% of unknown nucleotides-(N) and reads with less than 60% of bases with a quality value (Q-value) > 20 were removed using FASTP (version 0.18.0) (Chen et al., 2018). Paired end clean reads were merged as raw tags using FLSAH (version 1.2.11) (Magoč and Salzberg, 2011) with a minimum overlap of 10 bp and mismatch error rates of 2%. Noisy sequences of raw tags were filtered by QIIME (version 1.9.1) (Caporaso et al., 2010) pipeline under specific filtering conditions (Bokulich et al., 2013) to obtain the high-quality clean tags. The filtering conditions were as follows: (1) break raw tags from the first low-quality base site where the number of bases in the continuous low-quality value (the default quality threshold is ≤ 3) reaches the set length (the default length is 3); (2) then, filter tags whose continuous high-quality base length was less than 75% of the tag length. The clean tags were searched against the reference database (http://drive5.com/uchime/uchime_download.html) to perform reference-based chimera checking using the UCHIME algorithm. After chimeric tags were removed, the final effective tags were used for further analysis.

### Statistical Analyses

Effective tags were clustered into operational taxonomic units (OTUs) with at least 97% identity using the UPARSE pipeline (Edgar, 2013). The tag sequence with the highest abundance was selected as the representative sequence within each cluster. The representative sequences were classified into organisms based on a naïve Bayesian model with the RDP classifier (Wang et al., 2007) using the SILVA database (Pruesse et al., 2007), and the confidence threshold values range from 0.8 to 1. For the analyses among groups, Venn diagram-based analyses were performed in the R project to identify unique and common family and genus (Chen and Boutros, 2011). The stacked bar plot of the community composition was visualized in R project ggplot2 package (Wickham and Chang, 2015). α Diversity indices were calculated with QIIME. Comparisons of the α indexes among three groups were performed with Kruskal-Wallis test and Tukey’s HSD test using the R project. The R project was also used to analyze the data based on principal coordinates analysis (PCoA) of unweighted UniFrac distances and for plotting the results. Welch’s t-test, ANOSIM analysis, and ROC curve analysis were performed using the R project, and the KEGG pathway analysis of the OTUs was inferred using Tax4Fun (Aßhauer et al., 2015).

## Results

### Comparisons of Microbial Diversity Among IM, CC, and Control Groups

**Figure 1:**
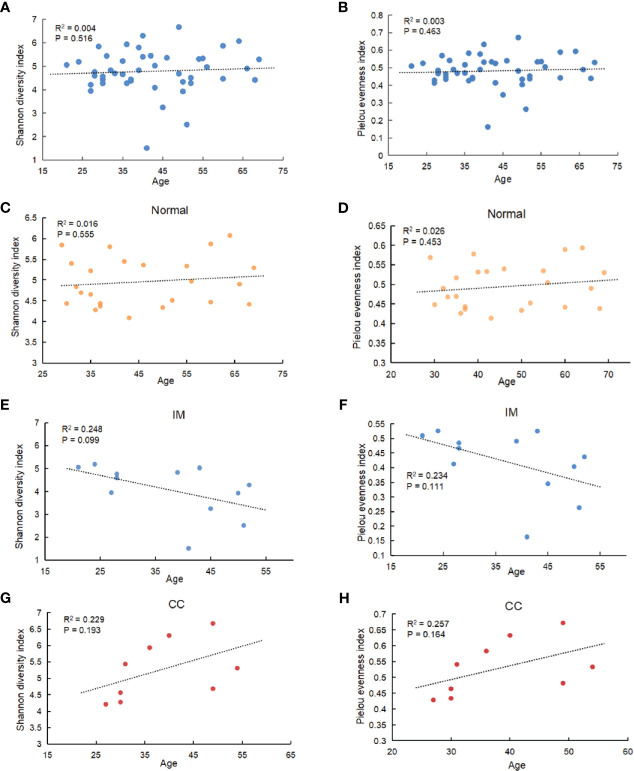
Correlation between gut microbiota and age. **(A)** Among all subjects with different ages, α diversity was determined by the Shannon diversity index. **(B)** Among all subjects with different ages, α diversity was determined by Pielou evenness index. **(C–F)** Correlation between gut microbiota and age in IM, CC, and control group separately (C: Shannon diversity index in Normal group; **(D)** Pielou evenness index in Normal group; **(E)** Shannon diversity index in IM group; **(F)** Pielou evenness index in IM group; **(G)** Shannon diversity index in CC group; **(H)** Pielou evenness index in CC group). Results showed that no age-related effect on gut microbiota in each group.

Fecal samples were collected from 12 patients with IM, nine patients with CC, and 24 healthy females. Clinical information is shown in **Table 1**. The V3 to V4 region of 16s rDNA was amplified and sequenced using Illumina Novaseq 6000 high-throughput sequencing platform. After quality filtering, a total of 4,644,583 16s rDNA genes (from 45 subjects) were identified for subsequent analysis. The average sequencing depth was 122,739 (95,472–135,073) reads per sample. Sequencing depth (Good’s coverage) was > 99%, which was sufficient for microbiota analysis in the IM, CC, and control groups. A total of 7,442 OTUs (97% identity) were observed across all samples. To remove the effect of age on gut microbiota, the correlation between gut microbiota and age was first analyzed in all samples and in each group separately. As indicated by Shannon diversity index and pielou evenness index (**Figures 1A, B**), there was no correlation between gut microbiota and age in all samples and in each group (**Figures 1C–H**).

**Table 1:** Summary of clinical information in each group and statistical analysis.

Characteristics	IM group	CC group	Control group	P value		
				IM *vs*. Normal	CC *vs*. Normal	CC *vs*. IM
Ethnicity	East Asia	East Asia	East Asia	/	/	/
Age range (mean ± se)	21 - 52 (37 ± 10)	27 – 54 (38 ± 11)	29 - 69 (40 ± 13)	0.099	0.219	0.980
β-HCG (mean ± se, mIU/ml)	113 - 41711 (9440 ± 1543)	7893 – 499816 (111903 ± 1550)	/	/	/	0.083
TNM stage	III (100%)	I (44.44%), III (55.56%)	/	/	/	/

For age range, mean, standard error and P value are indicated.

**Figure 2:**
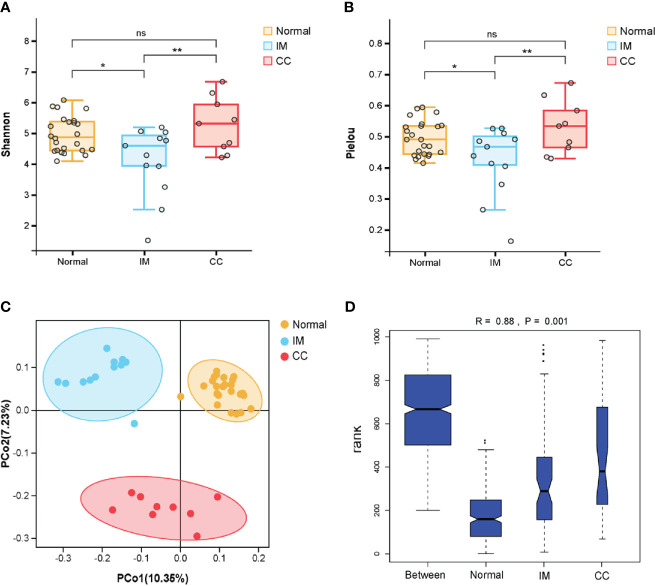
Comparisons of the Microbial Diversity among the IM, CC, and control groups. **(A–D)** Subjects were divided into three groups, i.e., Normal group (N = 24), IM group (N = 12), and CC group (N = 9). **(A, B)** As indicated by Shannon diversity index and Pielou evenness index, α-diversity of IM patients was significantly lower than that of healthy females as well as that of CC subjects. “*” means P value < 0.05; “**” means P value < 0.01 and “NS” means P value > 0.05. **(C)** Principle coordinate analysis (PCoA) of unweighted Unifrac distances among the three groups. Points represent samples. The samples in each group were ordinated closely respectively. **(D)** As tested by ANOSIM, the groups showed significant differences in similarity (R = 0.88, P_ANOSIM_ = 0.001).

The diversity of different taxa was analyzed by intra-group comparison (α diversity) and inter-group comparison (β diversity). Shannon diversity index and pielou evenness index were calculated with QIIME. As shown in **Figures 2A, B**, both Shannon diversity index (P_Kruskal-Wallis_ = 0.035) and pielou evenness index (P_Kruskal-Wallis_ = 0.045) were significantly different among groups. Specifically, Shannon diversity index in IM group was significantly lower than that in control group (P_TukeyHSD_ = 0.011). On average, the α diversity of CC group was slightly higher than that of control group, but the differences were not statistically significant (P_TukeyHSD_ =0.613). Shannon diversity index in CC group was higher than that in IM group (P_TukeyHSD_ =0.006). Pielou evenness index was similar to Shannon diversity index. The evenness of IM group was significantly lower than that of control group (P_TukeyHSD_ = 0.028). On average, the evenness of CC group was slightly higher than that of control group (P_TukeyHSD_ = 0.503), but this was not statistically significant. The evenness of CC group was significantly higher than that of IM group (P_TukeyHSD_ =0.008). Taken together, α diversity in IM group was significantly lower than that in control group, and species α diversity in CC group was significantly higher than that in IM group. Given these findings, principle coordinate analysis (PCoA) of unweighted Unifrac distances was used to estimate β diversity of gut microbiota among groups. The samples in each group were ordinated closely respectively, while the samples among three groups were separated obviously, indicating differences in bacterial structure among the groups (**Figure 2C**). Statistical analysis results of the differences among the three groups are shown in **Table 2**. Analysis of Similarity (ANOSIM) assessed by unweighted UniFrac demonstrated that there were significant differences in gut microbiota among IM, CC, and control groups at community level (R = 0.88, P = 0.001, **Figure 2D**).

**Table 2:** Beta diversity analysis and statistical analysis.

Group	IM *vs* Normal	CC *vs* Normal	CC *vs* IM
P value	0.001	0.001	0.042

P value for β diversity analysis is indicated.

### Bacterial Populations and Dominant Microbiome in Fecal Samples

**Figure 3:**
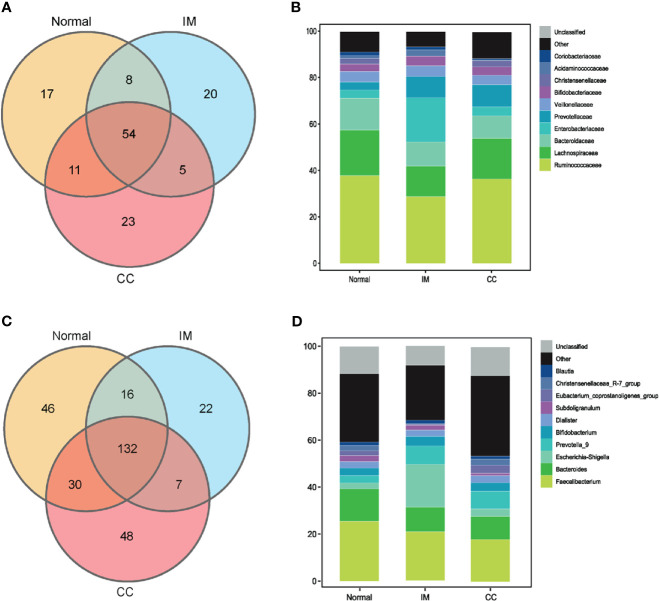
Bacterial populations and dominant microbiome in fecal samples. **(A)** Families in each group were examined for common and unique family using a Venn diagram. Overlapping areas represent common family between groups. **(B)** A cylindrical accumulation map at the family level. The relative abundance of the 10 most abundant bacteria in subjects. **(C)** Genus in each group were examined for common and unique genus using a Venn diagram. Overlapping areas represent common genus between different groups. **(D)** A cylindrical accumulation map at the genus level. The relative abundance of the 10 most abundant bacteria in subjects.

To understand the bacterial populations crossover among groups, we used a Venn diagram to indicate the differences among groups according to information on the gut microbiota abundance. The abundance of gut microbiota in patients with IM was lower than that in healthy controls at both the family and genus levels (**Figures 3A, C** **)**. Compared with IM patients and healthy controls, the abundance of unique gut microbiota in CC patients increased at both family level and genus level (**Figures 3A, C** **)**. Simultaneously, the two sets of data showed that the abundance of gut microbiota in IM patients was lower than that in controls, whereas the abundance of gut microbiota in CC patients was higher than that in controls and IM patients.

At the family level, the top 10 families were Ruminococcaceae, Lachnospiraceae, Bacteroidaceae, Enterobacteriaceae, Prevotellaceae, Veillonellaceae, Bifidobacteriaceae, Christensenellaceae, Acidaminococcaceae, and Coriobacteriaceae (**Figure 3B**). At the genus level, the top 10 genera were Faecalibacterium, Bacteroides, Escherichia-Shigella, Prevotella_9, Bifidobacterium, Dialister, Subdoligranulum, Eubacterium_coprostanoligenes_group, Christensenellaceae_R-7_group, and Blautia, respectively. In this study, the common bacteria, such as Faecalibacterium, Bacteroides, Escherichia-Shigella, Prevotella_9, and Bifidobacterium, were mainly found in each group (**Figure 3D**).

### Predicted Functional Changes in Microbiomes Among IM, CC, and Control Groups

**Figure 4:**
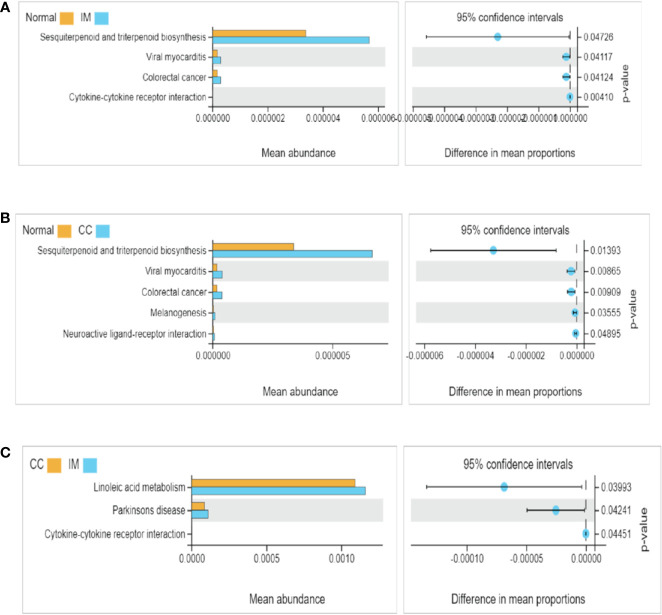
Functional pathway analysis of gut microbiome among IM, CC, and control groups. Microbial functions were predicted using Tax4Fun at the third level of the KEGG pathway, and statistically analyzed by Welch’s t-test between groups. KEGG pathways with significant abundance difference (P < 0.05) are shown. **(A)** Differential functional pathways between IM subjects and healthy females. **(B)** Differential functional pathways between CC subjects and healthy females. **(C)** Differential functional pathways between CC subjects and IM subjects. In each of the figures, the left panel shows the abundance of pathway differences between groups, and each bar in the graph represents the mean of each pathway in each group with significant differences in abundance between the groups. The graph on the right shows the difference between the confidence levels of the groups. The leftmost endpoint of each circle in the figure represents the 95% confidence interval lower limit of the mean difference, and the rightmost endpoint of the circle represents the 95% confidence interval upper limit of the mean difference. The center of the circle represents the difference in the mean. The group represented by the circle color is a group with a higher mean. At the far right of the displayed results is the inter-group significance Welch’s t-test p-value for the corresponding pathway.

The functional differences of gut microbiota among the three groups were predicted and compared. 16S rDNA sequencing data were analyzed using Tax4Fun, and a total of 284 KEGG pathways were generated. Analysis of fecal samples showed that compared with controls, higher abundance of gut microbiota in IM subjects was associated with pathways involved in sesquiterpenoid and triterpenoid biosynthesis, viral myocarditis, and colorectal cancer (**Figure 4A**). Surprisingly, compared with controls, higher abundance of gut microbiota in CC subjects was associated with pathways involved in melanogenesis and neuroactive ligand-receptor in addition to the above three pathways (**Figure 4B**). Additionally, compared with IM subjects, lower abundance of gut microbiota in CC subjects was associated with pathways involved in linoleic acid metabolism, Parkinson’s disease, and cytokine-cytokine receptor (**Figure 4C**).

### Changes in Bacterial Composition Among IM, CC, and Control Groups

**Figure 5:**
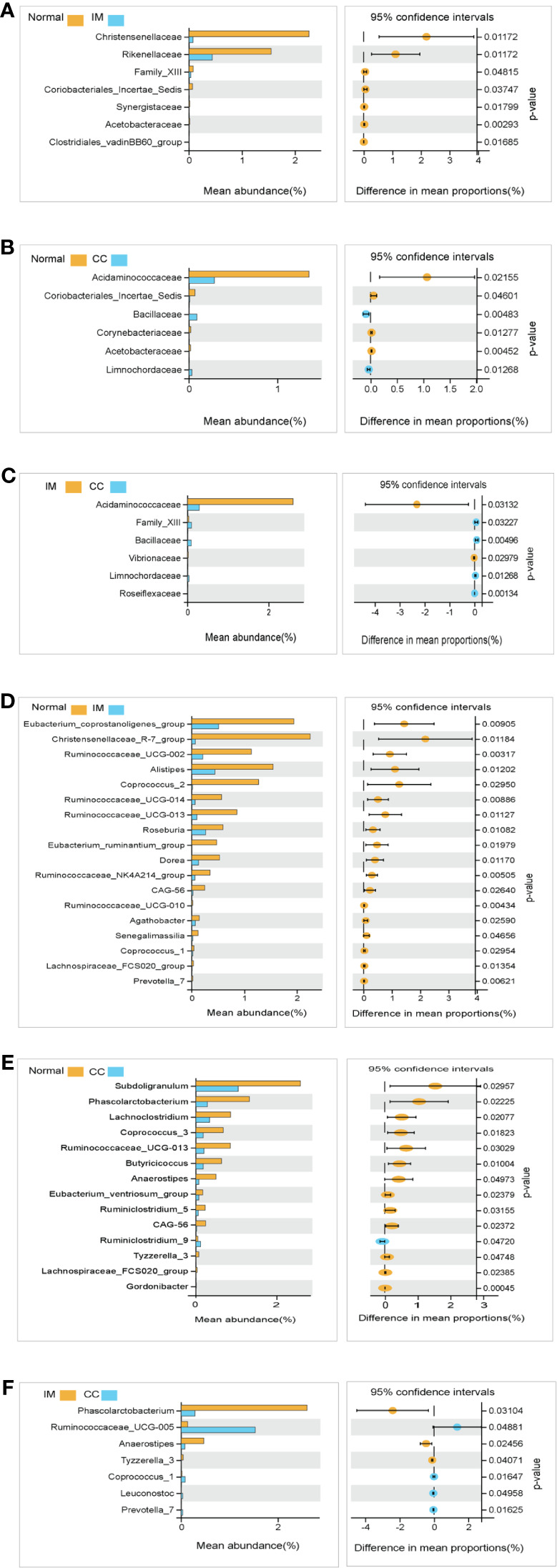
Species analysis of differences between groups analyzed by Welch’s t-test. **(A)** Differential species between healthy females and IM subjects at the family levels. **(B)** Differential species between healthy females and CC subjects at the family levels. **(C)** Differential species between IM subjects and CC subjects at the family levels. **(D)** Differential species between healthy female subjects and IM subjects at the genus levels. **(E)** Differential species between healthy female subjects and CC subjects at the genus levels. **(F)** Differential species between CC subjects and IM subjects at the genus levels. In each figure, the left panel shows the abundance of differences in species between groups, and each bar in the graph represents the mean of each species in each group, with significant differences in abundance between groups. The graph on the right shows the difference in confidence levels between groups. The leftmost endpoint of each circle in the figure represents the 95% confidence interval lower limit of the mean difference, and the rightmost endpoint of the circle represents the 95% confidence interval upper limit of the mean difference. The center of the circle represents the difference in the mean. The group represented by the circle color is a group with a higher mean. At the far right of the displayed results is the inter-group significance Welch’s t-test p-value for the corresponding species.

We compared the gut microbiota profiles in each group. At the family level, compared with the control group, the microbial abundance of seven families in IM subjects was significantly reduced, namely *Christensenellaceae*, *Rikenellaceae*, *Family_XIII*, *Coriobacteriales_Incertae_Sedis*, *Synergistaceae*, *Acetobacteraceae*, and *Clostridiales_vadinBB60_group* (**Figure 5A**), whereas in CC subjects, the microbial abundance was decreased significantly in four families (*Acidaminococcaceae*, *Coriobacteriales_Incertae_Sedis*, *Corynebacteriaceae*, and *Acetobacteraceae*), but increased in the other two families (*Bacillaceae* and *Limnochordaceae*) (**Figure 5B**). Notably, compared with IM subjects, the microbial abundance in CC subjects was decreased significantly in two families (*Acidaminococcaceae* and *Vibrionaceae*), but increased in the other four families (*Family_XIII*, *Bacillaceae*, *Limnochordaceae*, and *Roseiflexaceae*) (**Figure 5C**).

**Figure 6:**
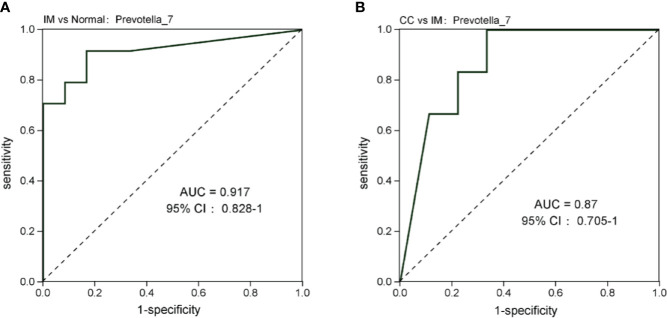
*Prevotella_7* is able to discriminate IM group from Normal group and CC group. **(A)** ROC curve analysis of IM group and Normal group. **(B)** ROC analysis of CC group and IM group.

At the genus level, compared with the control group, the microbial abundance of 21 genera in IM subjects was significantly reduced, and the top 5 differed microbes were *Eubacterium_coprostanoligenes_group*, *Christensenellaceae_R-7_group*, *Ruminococcaceae_UCG-002*, *Alistipes*, and *Coprococcus_2* (**Figure 5D**), respectively, whereas in the CC subjects, the microbial abundance were significantly decreased in 14 genera. The top 5 strains were *Subdoligranulum*, *Phascolarctobacterium*, *Lachnoclostridium*, *Coprococcus_3*, and *Ruminococcaceae_UCG-013* (**Figure 5E**). However, in CC subjects, the microbial abundance was significantly decreased in three genera (*Phascolarctobacterium*, *Anaerostipes*, and *Tyzzerella_3*), but significantly increased in four genera (*Ruminococcaceae_UCG-005*, *Coprococcus_1*, *Leuconostoc, and Prevotella_7*), compared with those in IM subjects (**Figure 5F**). In those significantly altered genera, the abundance of *Prevotella_7* showed the most significant difference not only between IM subjects and normal healthy control but also between CC subjects and IM subjects. To evaluate whether *Prevotella_7* could be used to distinguish IM subjects from normal healthy control or CC patients, ROC analysis was performed for *Prevotella_7*, and it yielded a ROC curve value of 0.917 (95% CI: 0.828-1) with an 91.7% sensitivity and 83.3% specificity in distinguishing IM patients from controls (**Figure 6A**). Meanwhile, *Prevotella_7* yielded a ROC curve value of 0.87 (95% CI: 0.705-1) with a 100% sensitivity and 66.7% specificity in distinguishing CC patients from IM patients (**Figure 6B**).

## Discussion

GTN is a malignant tumor with four phenotypes: IM, CC, PSTT, and ETT (Mangili et al., 2014). Accurate diagnosis leads to a high cure rate of 98% for CC and IM patients (Abu-Rustum et al., 2019). Therefore, this study aims to find novel biomarkers for CC and IM. Tumor microenvironment (TME) is closely related to the genesis and development of tumors (Arneth, 2019). Biological disorders of intestine can reshape TME, making it conducive to tumor growth (Shi et al., 2020). In various cancers (Meng et al., 2018; Shi et al., 2020), gut microbiota (bacteria, viruses, protozoa, archaea, and fungi in the gastrointestinal tract) could cause cellular DNA damage, host immune response (Rooks and Garrett, 2016), and chronic inflammation (Saad et al., 2016). However, to date, no studies have been conducted on the role of gut microbiota in GTN. In this study, we characterized the gut microbiota of female patients with IM or CC and healthy females by 16s rDNA sequencing. The results showed that there were significant differences in α and β diversity between IM, CC, and the control group, indicating differences in the composition of gut microbiota.

The abundance and diversity of gut microbiome have significant effects on carcinogenesis and immune response (McKenzie et al., 2021). Travis T. Sims et al. compared the fecal microbiome of 42 cervical cancer patients with 46 healthy female controls, and found that the α diversity was significantly higher in cervical cancer patients, and that the β diversity was significantly different across health status (Sims et al., 2019). Similarly, Wang et al. compared the gut microbiome of eight cervical cancer patients and five healthy controls. Unfortunately, in patients with cervical cancer, the α diversity of gut microbiota showed an increasing trend, but the difference was not statistically significant, whereas β diversity showed a significant difference (Wang et al., 2019). Notably, our study firstly revealed the fecal microbiome of patients with GTN (IM and CC). 16s rDNAs in the feces of patients with IM and CC were measured and compared pairwise with those of healthy controls. The results showed that the α diversity of the IM group was significantly lower than that of the control group, and with respect to β-diversity, there was a clear separation among the three groups, confirming compositional differences in the gut microbiota according to health status. Zhang et al. and Rivero-Segura N A et al. both found that there was a correlation between age and human gut microbiota (Rivero-Segura et al., 2020). Similarly, Jeffery et al. found that advanced age is associated with variations in the composition of gut microbiome characterized by a loss of diversity in specific taxa (Jeffery et al., 2016). Moreover, Claesson et al. found that individuals over 65 years of age were more likely to display a loss of taxa associated with diversity (Claesson et al., 2011). Different from the above studies, our study showed that there was no correlation between age and the richness and diversity of gut microbiome, suggesting that the diversity difference of these 45 samples was not related to age, but related to the different health status (IM or CC).

Dysbiosis of gut microbiota may be implicated in carcinogenesis, therapy-related side effects, and treatment outcomes. In particular, microbiome is involved in the modulation of inflammation and metabolism, thus affecting the occurrence and development of tumors (Hanahan and Weinberg, 2011). After unmasking the significant differences in the α and β diversity of gut microbiota in the feces of IM patients, CC patients, and health women, we further investigated the potential role of gut microbiome based on KEGG pathways. Interestingly, we found that the relative abundance of colorectal cancer-related genes was significantly increased in the IM and CC groups compared with the normal groups, suggesting that IM and CC might be associated with colorectal cancer-related genes. Further, we analyzed the composition of gut microbes in patients with IM and CC. Our results found that the microbial composition in each group differed significantly at both family level and genus level (19 families and 39 genera). At the family level, the *Christensenellaceae* family (a family recently described Firmicutes) plays an important role in human health, and the relative abundance of *Christensenellaceae* in the intestines of patients with metabolic diseases and inflammation is significantly reduced (Waters and Ley, 2019). Consistently, our study showed that the abundance of *Christensenellaceae* was significantly reduced in the IM compared with the control group, suggesting that *Christensenellaceae* may be involved in the development of IM *via* metabolism-related pathways. However, the specific molecular mechanism remains unclear, and further study is required. Besides, the gut microbiome may affect the severity of psychoneurological symptoms (PNS) related to cancer treatments *via* neural, immune, and endocrine signaling pathways. Bai Jinbing et al. found that the abundance of *Acidaminococcaceae* was higher in head and neck cancer patients with low PNS (Bai et al., 2020). Similarity, we found a decrease in the abundance of Acidaminococcaceae in patients with IM and CC compared with healthy controls. In addition, our reports, coupled with Bai Jinbing’s findings, implied a plausible role of *Acidaminococcaceae* in IM and CC. Jacqueline et al. identified the association between the structure of gut microbiota and the eye disc tumor in Drosophila larvae. In addition, they also found that the relative abundance of *Bacillaceae* in cancer-forming larvae was much lower than that in non-tumor–forming individuals, which may be related to the efficiency of the immune system and the immunogenicity of *Bacillaceae* lipopolysaccharides (Vatanen et al., 2016; Jacqueline et al., 2017). In this study, the abundance of *Bacillaceae* in CC patients was increased compared with healthy controls and IM patients, suggesting that CC patients had higher demands on the immune system.

There were also significant differences in the genus level between groups, with the abundance of most microbes in patients with IM and CC being lower than those in healthy women. *Prevotella_7* was the microorganism with the most significant variation in abundance between IM patients and healthy controls, as well as between CC patients and IM patients. The relative abundance of *Prevotella_7* in the feces of IM patients was significantly lower than that of both healthy women and CC patients, whereas the relative abundance of *Prevotella_7* in the feces of CC patients was slightly higher, but still not statistically significant compared with that of healthy women. ROC curve analysis demonstrated *Prevotella_7* might be as a biomarker in distinguishing IM patients from both normal healthy controls and CC patients, with high sensitivity and high specificity. Currently, the diagnosis strategies for GTN include patient review, β-hCG measurement and staging with (Doppler) ultrasonography (US) pelvis, and chest X-ray (CXR), in which β-hCG measurement is the primary method, but continuous measurement may take weeks or even months, and chest X-ray (CXR) would cause radiation to the human body. Obviously, gut microbiome is a novel diagnostic tool that takes less time and is harmless to humans. Besides, it is still a challenge to have accurate diagnosis of IM or CC based on current diagnosis methods because of the very similar clinical features of IM and CC. Interestingly, we found that *Prevotella_7* might be a novel biomarker in distinguishing IM patients from CC patients. Previous studies have shown that the relative abundance of *Prevotella* at mucosal sites is associated with various inflammatory diseases, including bacterial vaginosis, periodontitis, and rheumatoid arthritis (Scher et al., 2013; Anahtar et al., 2015). Studies suggested that *Prevotella* rich environments stimulate dendritic cells (DCs) to release interleukin-1β (IL-1β), IL-6, and IL-23, *via* toll like receptor 2, thereby IL-17 production by T helper 17 cells and neutrophils activation (Berezow and Darveau, 2000). Therefore, we suggested the role of *Prevotella_7* in host immunity may also be related to the risk and treatment outcome of IM and CC. However, the findings in the present study were based on the limited samples with no further functional experiments, the effect of altered abundance of *Prevotella_7* and other microbiota (such as *Christensenellaceae, Bacillaceae*, and *Christensenellaceae*) on the development of tumorigenesis as well as the impact of tumor development on the abundance of microbial still require further investigation.

In conclusion, our study revealed the fecal microbiome of GTN for the first time. α diversity, β diversity, relevant pathways, and microbial composition were analyzed by measuring 16s rDNA of the gut microbiome in the feces of IM patients, CC patients, and healthy women. Our study demonstrated distinct differences in the diversity and microbial composition of the gut microbiota among groups. Alteration of *Prevotella_7* may be used to predict the occurrence and disease progression of GTN. Our results provided new lights on the pathogenic mechanism of GTN, as well as novel therapeutic targets for the diagnosis and treatment of GTN.

## Data Availability Statement

The datasets presented in this study can be found in online repositories. The names of the repository/repositories and accession number(s) can be found below: https://www.ncbi.nlm.nih.gov/, SRP313846.

## Ethics Statement

The studies involving human participants were reviewed and approved by Medical Ethical Committee of Shengjing Hospital Affiliated to China Medical University. The patients/participants provided their written informed consent to participate in this study.

## Author ContributionS

Subject identification and data collection were performed by all authors. Besides, all authors participated in the interpretation of statistical analysis, and review and approval of final manuscript. Study concept was developed by XL and XM. Drafting of manuscript was done by XL. All authors contributed to the article and approved the submitted version.

## Funding

The study was supported by the “Revitalizing Liaoning Talents Plan” of Liaoning Province (No. XLYC1902003), National Natural Science Foundation of China (No. 81872123), Program for Liaoning Innovative Talents in University, “Major Special Construction Plan” for Discipline Construction of China Medical University in 2018 (No. 3110118029), Distinguished Professor of Liaoning Province, the Outstanding Scientifific Fund of Shengjing Hospital (No. 201601).

## Conflict of Interest

The authors declare that the research was conducted in the absence of any commercial or financial relationships that could be construed as a potential conflict of interest.

## Publisher’s Note

All claims expressed in this article are solely those of the authors and do not necessarily represent those of their affiliated organizations, or those of the publisher, the editors and the reviewers. Any product that may be evaluated in this article, or claim that may be made by its manufacturer, is not guaranteed or endorsed by the publisher.

## References

[B1] Aßhauer K. P. Wemheuer B. Daniel R. Meinicke P. (2015). Tax4Fun: Predicting Functional Profiles From Metagenomic 16S rRNA Data. Bioinf. (Oxford England) 31, 2882–2884. 10.1093/bioinformatics/btv287 PMC4547618 25957349

[B2] Abu-Rustum N. R. Yashar C. M. Bean S. Bradley K. Campos S. M. Chon H. S.. (2019). Gestational Trophoblastic Neoplasia, Version 2.2019, NCCN Clinical Practice Guidelines in Oncology. J. Natl. Compr. Cancer Network: JNCCN 17, 1374–1391. 10.6004/jnccn.2019.0053 31693991

[B3] AlHilli M. M. Bae-Jump V. (2020). Diet and Gut Microbiome Interactions in Gynecologic Cancer. Gynecol Oncol. 159, 299–308. 10.1016/j.ygyno.2020.08.027 32933758

[B4] Anahtar M. N. Byrne E. H. Doherty K. E. Bowman B. A. Yamamoto H. S. Soumillon M.. (2015). Cervicovaginal Bacteria are a Major Modulator of Host Inflammatory Responses in the Female Genital Tract. Immunity 42, 965–976. 10.1016/j.immuni.2015.04.019 25992865 PMC4461369

[B5] Arneth B. (2019). Tumor Microenvironment. Med (Kaunas Lithuania) 56, 15. 10.3390/medicina56010015 PMC7023392 31906017

[B6] Bai J. Bruner D. W. Fedirko V. Beitler J. J. Zhou C. Gu J.. (2020). Gut Microbiome Associated With the Psychoneurological Symptom Cluster in Patients With Head and Neck Cancers. Cancers (Basel) 12. 10.3390/cancers12092531 PMC7563252 32899975

[B7] Berezow A. B. Darveau R. P. (2000). Microbial Shift and Periodontitis. Periodontology 2011, 55:36–47. 10.1111/j.1600-0757.2010.00350.x PMC3058494 21134227

[B8] Bokulich N. A. Subramanian S. Faith J. J. Gevers D. Gordon J. I. Knight R.. (2013). Quality-Filtering Vastly Improves Diversity Estimates From Illumina Amplicon Sequencing. Nat. Methods 10, 57–59. 10.1038/nmeth.2276 23202435 PMC3531572

[B9] Brestoff J. R. Artis D. (2013). Commensal Bacteria at the Interface of Host Metabolism and the Immune System. Nat. Immunol. 14, 676–684. 10.1038/ni.2640 23778795 PMC4013146

[B10] Caporaso J. G. Kuczynski J. Stombaugh J. Bittinger K. Bushman F. D. Costello E. K.. (2010). QIIME Allows Analysis of High-Throughput Community Sequencing Data. Nat. Methods 7, 335–336. 10.1038/nmeth.f.303 20383131 PMC3156573

[B11] Chase D. Goulder A. Zenhausern F. Monk B. Herbst-Kralovetz M. (2015). The Vaginal and Gastrointestinal Microbiomes in Gynecologic Cancers: a Review of Applications in Etiology, Symptoms and Treatment. Gynecol. Oncol. 138, 190–200. 10.1016/j.ygyno.2015.04.036 25957158

[B12] Chen H. Boutros P. C. (2011). VennDiagram: a Package for the Generation of Highly-Customizable Venn and Euler Diagrams in R. BMC Bioinf. 12, 35. 10.1186/1471-2105-12-35 PMC3041657 21269502

[B13] Chen S. Zhou Y. Chen Y. Gu J. (2018). Fastp: an Ultra-Fast All-in-One FASTQ Preprocessor. Bioinf. (Oxford England) 34, i884–ii90. 10.1093/bioinformatics/bty560 PMC6129281 30423086

[B14] Claesson M. J. Cusack S. O’Sullivan O. Greene-Diniz R. de Weerd H. Flannery E.. (2011). Composition, Variability, and Temporal Stability of the Intestinal Microbiota of the Elderly. Proc. Natl. Acad. Sci. U. S. A. 108 (Suppl 1), 4586–4591. 10.1073/pnas.1000097107 20571116 PMC3063589

[B15] Edgar R. C. (2013). UPARSE: Highly Accurate OTU Sequences From Microbial Amplicon Reads. Nat. Methods 10, 996–998. 10.1038/nmeth.2604 23955772

[B16] FIGO Committee on Gynecologic Oncology. (2009). Current FIGO Staging for Cancer of the Vagina, Fallopian Tube, Ovary, and Gestational Trophoblastic Neoplasia. Int. J. Gynaecol Obstetrics 105, 3–4. 10.1016/j.ijgo.2008.12.015 19322933

[B17] Golfier F. Raudrant D. Frappart L. Mathian B. Guastalla J. P. Trillet-Lenoir V.. (2007). First Epidemiological Data From the French Trophoblastic Disease Reference Center. Am. J. Obstetrics Gynecolo 196, 172.e1–172.e5. 10.1016/j.ajog.2006.10.867 17306669

[B18] Gopalakrishnan V. Helmink B. A. Spencer C. N. Reuben A. Wargo J. A. (2018). The Influence of the Gut Microbiome on Cancer, Immunity, and Cancer Immunotherapy. Cancer Cell 33, 570–580. 10.1016/j.ccell.2018.03.015 29634945 PMC6529202

[B19] Guo M. Wu F. Hao G. Qi Q. Li R. Li N.. (2017). Bacillus Subtilis Improves Immunity and Disease Resistance in Rabbits. Front. Immunol. 8, 354. 10.3389/fimmu.2017.00354 28424690 PMC5372816

[B20] Hanahan D. Weinberg R. A. (2011). Hallmarks of Cancer: The Next Generation. Cell 144, 646–674. 10.1016/j.cell.2011.02.013 21376230

[B21] Herrera S. Martínez-Sanz J. Serrano-Villar S. H. I. V. (2019). Cancer, and the Microbiota: Common Pathways Influencing Different Diseases. Front. Immunol. 10, 1466. 10.3389/fimmu.2019.01466 31316514 PMC6610485

[B22] Jacqueline C. Brazier L. Faugère D. Renaud F. Thomas F. Roche B. (2017). Can Intestinal Microbiota be Associated With non-Intestinal Cancers? Sci. Rep. 7, 12722. 10.1038/s41598-017-11644-9 28983086 PMC5629204

[B23] Jeffery I. B. Lynch D. B. O’Toole P. W. (2016). Composition and Temporal Stability of the Gut Microbiota in Older Persons. ISME J. 10, 170–182. 10.1038/ismej.2015.88 26090993 PMC4681863

[B24] Le Chatelier E. Nielsen T. Qin J. Prifti E. Hildebrand F. Falony G.. (2013). Richness of Human Gut Microbiome Correlates With Metabolic Markers. Nature 500, 541–546. 10.1038/nature12506 23985870

[B25] Lok C. Frijstein M. van Trommel N. (2020). Clinical Presentation and Diagnosis of Gestational Trophoblastic Disease. Best Pract. Res. Clin. Obstetrics Gynaecol. 74, 42–52 10.1016/j.bpobgyn.2020.12.001 33422446

[B26] Lurain J. R. (2010). Gestational Trophoblastic Disease I: Epidemiology, Pathology, Clinical Presentation and Diagnosis of Gestational Trophoblastic Disease, and Management of Hydatidiform Mole. Am. J. Obstetrics Gynecol. 203, 531–539. 10.1016/j.ajog.2010.06.073 20728069

[B27] Magoč T. Salzberg S. L. (2011). FLASH: Fast Length Adjustment of Short Reads to Improve Genome Assemblies. Bioinf. (Oxford England) 27, 2957–2963. 10.1093/bioinformatics/btr507 PMC3198573 21903629

[B28] Mangili G. Lorusso D. Brown J. Pfisterer J. Massuger L. Vaughan M.. (2014). Trophoblastic Disease Review for Diagnosis and Management: A Joint Report From the International Society for the Study of Trophoblastic Disease, European Organisation for the Treatment of Trophoblastic Disease, and the Gynecologic Cancer InterGroup. Int. J. Gynecol. Cancer 24, S109–S116. 10.1097/IGC.0000000000000294 25341573

[B29] McKenzie N. D. Hong H. Ahmad S. Holloway R. W. (2021). The Gut Microbiome and Cancer Immunotherapeutics: a Review of Emerging Data and Implications for Future Gynecologic Cancer Research. Crit. Rev. Oncol./Hematol. 157, 103165. 10.1016/j.critrevonc.2020.103165 33227575

[B30] McQuade J. L. Daniel C. R. Helmink B. A. Wargo J. A. (2019). Modulating the Microbiome to Improve Therapeutic Response in Cancer. Lancet Oncol. 20, e77–e91. 10.1016/S1470-2045(18)30952-5 30712808 PMC12908161

[B31] Meng C. Bai C. Brown T. D. Hood L. E. Tian Q. (2018). Human Gut Microbiota and Gastrointestinal Cancer. Genomics Proteomics Bioinf. 16, 33–49. 10.1016/j.gpb.2017.06.002 PMC6000254 29474889

[B32] Pruesse E. Quast C. Knittel K. Fuchs B. M. Ludwig W. Peplies J.. (2007). SILVA: a Comprehensive Online Resource for Quality Checked and Aligned Ribosomal RNA Sequence Data Compatible With ARB. Nucleic Acids Res. 35, 7188–7196. 10.1093/nar/gkm864 17947321 PMC2175337

[B33] Qian X. Liu Y. X. Ye X. Zheng W. Lv S. Mo M.. (2020). Gut Microbiota in Children With Juvenile Idiopathic Arthritis: Characteristics, Biomarker Identification, and Usefulness in Clinical Prediction. BMC Genomics 21, 286. 10.1186/s12864-020-6703-0 32264859 PMC7137182

[B34] Rivero-Segura N. A. Bello-Chavolla O. Y. Barrera-Vázquez O. S. Gutierrez-Robledo L. M. Gomez-Verjan J. C. (2020). Promising Biomarkers of Human Aging: In Search of a Multi-Omics Panel to Understand the Aging Process From a Multidimensional Perspective. Ageing Res. Rev. 64, 101164. 10.1016/j.arr.2020.101164 32977058

[B35] Rooks M. G. Garrett W. S. (2016). Gut Microbiota, Metabolites and Host Immunity. Nat. Rev. Immunol. 16, 341–352. 10.1038/nri.2016.42 27231050 PMC5541232

[B36] Saad M. J. Santos A. Prada P. O. (2016). Linking Gut Microbiota and Inflammation to Obesity and Insulin Resistance. Physiol. (Bethesda Md) 31, 283–293. 10.1152/physiol.00041.2015 27252163

[B37] Scher J. U. Sczesnak A. Longman R. S. Segata N. Ubeda C. Bielski C.. (2013). Expansion of Intestinal Prevotella Copri Correlates With Enhanced Susceptibility to Arthritis. eLife 2, e01202. 10.7554/eLife.01202 24192039 PMC3816614

[B38] Seckl M. J. Sebire N. J. Fisher R. A. Golfier F. Massuger L. Sessa C. (2013). Gestational Trophoblastic Disease: ESMO Clinical Practice Guidelines for Diagnosis, Treatment and Follow-Up. Ann. Oncol. 24 (Suppl 6), vi39–vi50. 10.1093/annonc/mdt345 23999759

[B39] Serino M. Luche E. Gres S. Baylac A. Bergé M. Cenac C.. (2012). Metabolic Adaptation to a High-Fat Diet Is Associated With a Change in the Gut Microbiota. Gut 61, 543–553. 10.1136/gutjnl-2011-301012 PMC3292714 22110050

[B40] Shi Y. Zheng W. Yang K. Harris K. G. Ni K. Xue L.. (2020). Intratumoral Accumulation of Gut Microbiota Facilitates CD47-Based Immunotherapy *via* STING Signaling. J. Exp. Med. 217, e20192282. 10.1084/jem.20192282 PMC7201921 32142585

[B41] Silva A. Monteiro K. D. N. Sun S. Y. Borbely A. U. (2021). Gestational Trophoblastic Neoplasia: Novelties and Challenges. Placenta S0143-4004, 00066-7. 10.1016/j.placenta.2021.02.013 33685753

[B42] Sims T. T. Colbert L. E. Zheng J. Delgado Medrano A. Y. Hoffman K. L. Ramondetta L.. (2019). Gut Microbial Diversity and Genus-Level Differences Identified in Cervical Cancer Patients Versus Healthy Controls. Gynecol Oncol. 155, 237–244. 10.1016/j.ygyno.2019.09.002 31500892 PMC6825899

[B43] Sivan A. Corrales L. Hubert N. Williams J. B. Aquino-Michaels K. Earley Z. M.. (2015). Commensal Bifidobacterium Promotes Antitumor Immunity and Facilitates Anti-PD-L1 Efficacy. Sci. (New York NY) 350, 1084–1089. 10.1126/science.aac4255 PMC4873287 26541606

[B44] Sun S. Y. Melamed A. Joseph N. T. Gockley A. A. Goldstein D. P. Bernstein M. R.. (2016). Clinical Presentation of Complete Hydatidiform Mole and Partial Hydatidiform Mole at a Regional Trophoblastic Disease Center in the United States Over the Past 2 Decades. Int. J. Gynecol. Cancer 26, 367–370. 10.1097/IGC.0000000000000608 26588240

[B45] Vatanen T. Kostic A. D. d’Hennezel E. Siljander H. Franzosa E. A. Yassour M.. (2016). Variation in Microbiome LPS Immunogenicity Contributes to Autoimmunity in Humans. Cell 165, 842–853. 10.1016/j.cell.2016.04.007 27133167 PMC4950857

[B46] Victoria Diniz M. Sun S. Y. (2018). Experience With the Use of an Online Community on Facebook for Brazilian Patients With Gestational Trophoblastic Disease: Netnography Study. J. Med. Internet Res. 20, e10897. 10.2196/10897 30249575 PMC6231805

[B47] Wang Q. Garrity G. M. Tiedje J. M. Cole J. R. (2007). Naive Bayesian Classifier for Rapid Assignment of rRNA Sequences Into the New Bacterial Taxonomy. Appl. Environ. Microbiol. 73, 5261–5267. 10.1128/AEM.00062-07 17586664 PMC1950982

[B48] Wang Z. Wang Q. Zhao J. Gong L. Zhang Y. Wang X.. (2019). Altered Diversity and Composition of the Gut Microbiome in Patients With Cervical Cancer. AMB Express 9, 40. 10.1186/s13568-019-0763-z 30904962 PMC6431307

[B49] Waters J. L. Ley R. E. (2019). The Human Gut Bacteria Christensenellaceae are Widespread, Heritable, and Associated With Health. BMC Biol. 17, 83. 10.1186/s12915-019-0699-4 31660948 PMC6819567

[B50] Wickham H. Chang W. (2015). An Implementation of the Grammar of Graphics in R: Ggplot.

